# Aggregating amyloid resources: A comprehensive review of databases on amyloid-like aggregation

**DOI:** 10.1016/j.csbj.2024.10.047

**Published:** 2024-10-29

**Authors:** Valentín Iglesias, Jarosław Chilimoniuk, Carlos Pintado-Grima, Oriol Bárcenas, Salvador Ventura, Michał Burdukiewicz

**Affiliations:** a Clinical Research Centre, Medical University of Białystok, Białystok, Poland; b Institut de Biotecnologia i de Biomedicina and Departament de Bioquímica i Biologia Molecular, Universitat Autònoma de Barcelona, Bellaterra, Barcelona, Spain; c Institute of Advanced Chemistry of Catalonia (IQAC), CSIC, Barcelona, Spain; d Hospital Universitari Parc Taulí, Institut d′Investigació i Innovació Parc Taulí (I3PT-CERCA), Universitat Autònoma de Barcelona, Sabadell, Spain; e Institute of Biotechnology, Life Sciences Center, Vilnius University, Vilnius, Lithuania

**Keywords:** Amyloid, Bioinformatics, Biotherapeutics, Database, Protein aggregation, Protein solubility, Prion

## Abstract

Protein aggregation is responsible for several degenerative conditions in humans, and it is also a bottleneck in industrial protein production and storage of biotherapeutics. Bioinformatics tools have been developed to predict and redesign protein solubility more efficiently by understanding the underlying principles behind aggregation. As more experimental data become available, dedicated resources for storing, indexing, classifying and consolidating experimental results have emerged. These resources vary in focus, including aggregation-prone regions, 3D patches or protein stretches capable of forming amyloid fibrils. Some of these resources also consider the experimental conditions that cause protein aggregation and how they affect the process. This review article explores how protein aggregation databases have evolved and surveys state-of-the-art resources. We highlight their applications, complementarity and existing limitations. Moreover, we showcase the existing symbiosis between amyloid-related databases and predictive tools. To increase the usefulness of our review, we supplement it with a comprehensive list of present and past amyloid databases: https://biogenies.info/amyloid-database-list/.

## 1 Introduction

Protein aggregation is a second-order reaction in which soluble monomeric species transit towards multimeric architectures forming protein deposits, usually highly ordered fibrillar structures named amyloids. The amyloid conformation consists of an intermolecular in-register stacking of β-stranded proteins in parallel or antiparallel form, which runs perpendicular to the fiber axis [1]. Amyloid fibrils can be detected by the binding of specific dyes such as Thioflavin-T or Congo Red, detergent and proteolytic resistance, and the presence of cross-β signals on X-ray diffraction patterns, typically at 4.7 and 10.2 Å [2]. The ability to form amyloid structures appears to be a generic property of proteins and is not tied to specific sequences of amino acids [3].

This fibrillization of proteins is widely recognized as a key factor in the onset of a myriad of different debilitating human conditions known as protein amyloidoses. This category covers disorders such as Parkinson's disease (PD), Alzheimer's disease (AD), Creutzfeldt-Jakob's, Amyotrophic Lateral Sclerosis (ALS) or Transthyretin (ATTR) amyloidosis [4]. Structural determinations of different amyloids from biopsies reveal the same core amyloid-forming protein can achieve multiple arrangements *in vivo* [5]. Specific amyloid polymorphs are thought to be associated with distinct disease manifestations [6]. The International Society of Amyloidosis recommends notating proteins constituting amyloid fibrils which deposition causes these disorders with an initial A (or amyloid) [7].

Recently, amyloidoses has garnered attention in the context of diabetes and various cancers, as well as viral, parasitic and bacterial infections [8], [9], [10], [11]. Moreover, amyloids can also indirectly accelerate the onset of other diseases through cross-seeding, where one type of amyloid aggregate promotes the self-assembly of a different type of amyloidogenic protein [12].

The aggregation of protein-based products is a significant concern in the biopharmaceutical industry. While not always amyloid-like, such aggregation can result in substantial economic losses, leading to production bottlenecks, particularly in developing biotherapeutics and other protein-based products [13]. Consequently, considerable effort has been devoted to minimizing aberrant self-assembly during the development, production, and formulation of these proteins [14].

The same properties of amyloids that lead to their pathological aggregation in cells, in turn, are beneficial for organisms to develop specific biological functions such as scaffolding bacterial biofilms, eukaryotic eggshells or serving as amino acid storage in plant seeds [15], [16], [17]. This phenomenon prompted efforts to generate amyloids of functionalized self-assembled nanostructures [18], [19]. Moreover, disturbing protein homeostasis by inducing protein aggregation has been shown as a viable antimicrobial strategy [20], [21].

A protein's aggregation propensity is primarily determined by its amino acid sequence and the spatial arrangement of such residues. Consecutive amino acids with high aggregation propensity constitute aggregation-prone regions (APRs). High hydrophobicity, low local or net charge and a favorable propensity to form a β-sheet structure are considered the primary factors contributing to amyloid aggregation of linear amyloid sequences [2], [6], [22]. Cryptic Amyloidogenic Regions (CARs) are sequential stretches found in disordered proteins with mild amyloidogenic character [23]. CARs are widespread in IDRs and other low-complexity regions such as PrLDs [24]. This is due to their lower risk of undergoing pathogenic aggregation while maintaining a high prevalence to establish functional protein-protein interactions.

On the other hand, folded proteins may display spatially clustered APRs (STAPs), including non-consecutive amino acids [5]. However, clustered hydrophobic amino acids protected from the solvent in the hydrophobic core or transmembrane segments have negligible effect on protein aggregation. Thus, knowledge of their 3D conformation is required to correctly assess folded proteins' aggregation potential in their native state. These sequential and structural elements, among others, dictate the amyloid-forming capabilities of proteins.

The protein aggregation propensity of a given polypeptide can be heavily modulated by environmental factors impacting the reaction's kinetics, thermodynamics or structural properties. Protein concentration, incubation temperature, pH, identity and osmolarity of salts, reducing/oxidizing compounds, post-translational modifications (PTMs), presence of lipids, presence or absence of pre-formed fibrils or other additives, as well as stirring the samples have a particular effect on the deposition process [25], [26].

One of the specific examples of amyloids is prion proteins. Prusiner first coined the term “prion” to refer to the proteinaceous particle capable of inducing neurodegenerative conditions in mammals [27]. This protein could post-translationally convert the soluble native state into an infectious, self-templating and self-propagating cytotoxic conformation between cells, individuals and even species [28]. Expansion of the prion phenomenon beyond mammalian diseases allowed the identification of novel prions and also of prion-like proteins and their prion-like domains (PrLDs): proteins capable of prion conversion but unable of transmission between individuals or species [29].

Due to the multifaceted complexity of amyloid aggregation, involving genetic, biochemical, and physicochemical factors, researchers attempted to gather knowledge on that topic in dedicated databases [30]. This review aims to list the currently available databases on that topic, present their scope and discuss their co-evolution with dedicated predictive algorithms. Moreover, we showcase how the field of amyloid self-assembly impacts the prediction and annotation of non-amyloid aggregation.

## 2 Amyloid aggregation databases

**Fig. 1:**
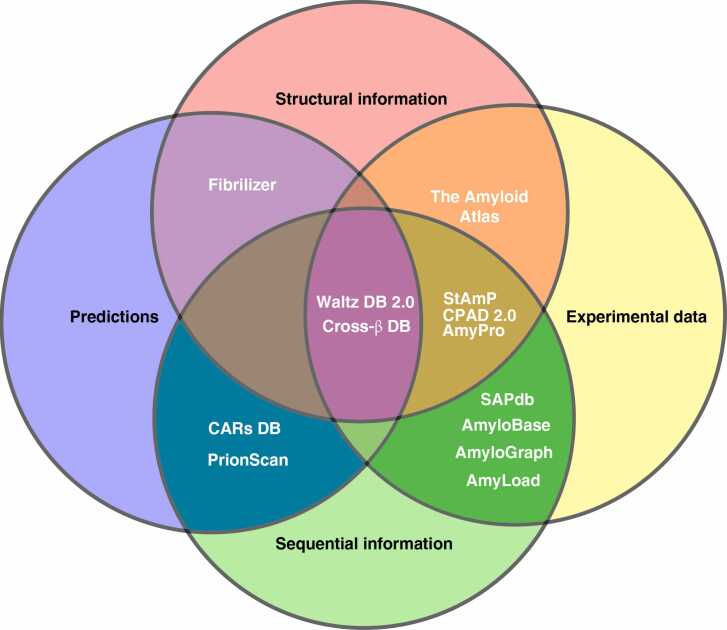
Summary of active amyloid databases. A distinction is made on the source of their information (experimental or predicted) and the level of structural complexity considered (sequences or structures).

In our analysis of amyloid databases, we categorize the available resources based on two primary criteria. Firstly, we differentiate between databases containing only information on amyloid sequences and those focusing on detailed structural data. Secondly, we classify these databases by the nature of their data, distinguishing between experimentally confirmed data and emerging datasets of predicted amyloid-related properties (**see** Fig. 1 and Table 1).

**Table 1:** Amyloid databases described in this manuscript. The interactive table with extended database descriptions is available online at**:**https://biogenies.info/amyloid-database-list/**.** Abbreviations used in the table: ENA: European Nucleotide Archive; CPAD: Curated Protein Aggregation Database; KEGG: Kyoto Encyclopedia of Genes and Genomes.

Database	Link to database	Reference	Data sources and links to other databases and software
AmyLoad	http://comprec-lin.iiar.pwr.edu.pl/amyload/	Wozniak and Kotulska [31]	TANGO; WALTZ; AmylFrag; AGGRESCAN; AmylHex; PubMed
AmyloBase	http://bioserver2.sbsc.unifi.it/AmyloBase.html	Belli, Ramazzotti, and Chiti [32]	PubMed; UniProt
AmyloGraph	http://amylograph.com/	Burdukiewicz et al. [33]	PubMed; UniProt
Amyloid Atlas	https://people.mbi.ucla.edu/sawaya/amyloidatlas/	Sawaya et al. [1]	PDB; PubMed
AmyPro	http://amypro.net	Varadi et al. [34]	PDB; UniProt; PubMed
CARs DB	http://carsdb.ppmclab.com/	Pintado-Grima, et al. [35]	DisProt; UniProt
CPAD 2.0	https://web.iitm.ac.in/bioinfo2/cpad2/index.html	Rawat et al. [36]	CPAD; Waltz-DB 2.0; AmyLoad; AmyPro; UniProt; PDB; PubMed
Cross-Beta DB	https://crossbetadb.crbm.cnrs.fr/	Gonay et al. [37]	PDB; AmyPro; UniProt; PubMed
Fibrilizer	https://amyloid.cs.mcgill.ca/database/index.html	Smaoui et al. [38]	PDB
PrionScan	http://webapps.bifi.es/prionscan/	Espinosa Angarica et al. [39]	UniProt; ENA; Protein; KEGG; Pfam; QuickGO
StAmP	https://stamp.switchlab.org/	Louros et al. [40]	WALTZ-DB; AmyPro; CPAD; PDB; UniProt
Waltz DB 2.0	http://waltzdb.switchlab.org/	Louros, Konstantoulea, et al. [41]	PubMed; UniProt; PDB

### 2.1 AmyLoad

AmyLoad (http://comprec-lin.iiar.pwr.edu.pl/amyload/)[31] collects peptides and proteins with experimentally verified amyloid propensity. The database stores 1400 entries with annotations on self-assembly potential and the experimental method and conditions employed to measure the aggregation.

### 2.2 AmyloBase

AmyloBase (http://bioserver2.sbsc.unifi.it/AmyloBase.html)[32] collects experimental data on the self-assembly kinetics of point mutants of three unique proteins and peptides. The database contains the studied fragment position in protein, its length and mass, protein origin, mutation type, number of hotspots, experimental conditions (pH, protein concentration, ionic strength, temperature), study method, the kinetics of aggregation (e.g., lag phase) and if the end product is amyloid fibrils.

### 2.3 AmyloGraph

AmyloGraph (http://amylograph.com/)[33] explores the concept of how different amyloid precursor proteins impact aggregation and amyloid formation of one another. The database stores manually curated data from 562 manuscripts, resulting in 896 records of experimentally derived data on amyloid-amyloid interactions for 46 amyloidogenic proteins. Each entry represents one interaction and can be studied as a graph or table. In the table format, users can find both interacting proteins' sequences, their length, impact on aggregation speed and fibril morphology (homo- or heterogeneous).

### 2.4 Amyloid atlas

Amyloid Atlas (https://people.mbi.ucla.edu/sawaya/amyloidatlas/)[1] provides a manually curated list of structures of human pathological amyloids based on cryogenic electron microscopy (cryo-EM), solid-state nuclear magnetic resonance (ssNMR) and microcrystal electron diffraction amyloid structures derived from PDB [42]. Storing 506 fibril entries at the moment of writing, Amyloid Atlas is the most comprehensive resource dedicated to the 3D structures of amyloids. Each entry contains the protein's name, origin, and the 3D structure colored according to the residue polarity and estimated solvation energy. Energetic stabilization is also itemized per chain, layer, and residue.

### 2.5 AmyPro

The AmyPro database (http://amypro.net)[34] covers 125 records categorized using four labels: functional amyloid, functional prion, pathogenic, biologically not relevant or not known. Each entry provides protein sequence with highlighted APRs. In the case of proteins with solved 3D structures, amyloid-forming stretches are marked on top. Moreover, AmyPro provides links to relevant publications describing amyloidogenicity of proteins.

### 2.6 Cryptic amyloidogenic regions database (CARs DB)

CARs-DB (http://carsdb.ppmclab.com/)[35] collects CARs within intrinsically disordered regions (IDRs), which are more polar and have milder aggregation potential than amyloidogenic stretches found in globular proteins. CARs-DB offers more than 8900 unique CARs across 1711 IDRs derived from the DisProt database [43]. Each CAR is described by its sequence, protein of origin, CAR position in the IDR and the Waltz score.

### 2.7 Curated protein aggregation database (CPAD) 2.0

CPAD 2.0 (https://web.iitm.ac.in/bioinfo2/cpad2/index.html)[36], [44] stores manually curated entries related to protein aggregation and is enriched with kinetic and structural information. The collection is divided into four databases: amyloidogenic peptides, APRs, aggregation kinetics, and structures of amyloid fibrils and amyloid-precursor proteins. The first one, peptide-centric, contains peptide sequence, its length, position in a sequence, origin, class (amyloid or non-amyloid), net and absolute charge, and results from NuAPRpred, TANGO [45], AGGRESCAN [46] and PASTA 2.0 [47]. The APR collection describes experimentally validated APRs, including peptide origin, category, mutation type, prion properties (if observed), APRs' protein position, length and sequence. The database of self-assembly kinetics gathers protein aggregation kinetic data, sequence type (wild or mutated), experimental conditions and measurement method. The database also features protein and peptide 3D structures, including the monomer length, origin, mutation type, class (amyloid or non-amyloid precursor), structure determination method, and resolution, if applicable.

### 2.8 Cross-beta DB

Cross-Beta DB (https://crossbetadb.crbm.cnrs.fr/)[37] gathers amyloid-forming regions of the naturally occurring cross-β structures within amyloid fibrils. The database is the result of careful manual curation on experimentally tested cross-β amyloid forming stretches. It contains 115 individual entries from 44 different amyloid-precursor proteins. In each entry, protein's origin, sequence, length, Archcandy2.0 [48] prediction results, experimental conditions (protein concentration, pH, temperature, buffer), measurement method and fibril state can be found. Moreover, the APR sequence, position, molecular weight and mutations, among others, are displayed. The experimentally obtained 3D structure can also be viewed alongside the amino acid composition graph.

### 2.9 Fibrillizer

Fibrillizer (https://amyloid.cs.mcgill.ca/database/index.html) collects results from CreateFibril [38], a tool that builds fibril atomic resolution models. The database focuses on energetically favorable amyloid fibril polymorphism, storing potential multiple combinations of energetically possible supra-fibrillar assemblies in the form of single, stack, ring and polygon structures for three proteins: Aβ42, Aamylin and HET-s.

### 2.10 Prionscan

PrionScan (http://webapps.bifi.es/prionscan/)[39] stores predicted prion-like proteins from complete proteomes. It stores approximately 28000 PrLDs for over 3200 organisms covering major taxonomic divisions. Each entry displays the protein's sequence and origin, and the predicted prion domain can be highlighted.

### 2.11 Structural analysis of Amyloid polymorphs (StAmP)

The StAmP (https://stamp.switchlab.org/)[40] database focuses on the structural diversity of amyloid fibril polymorphisms. The database, which results from manual curation and is enriched by automatic bioinformatic analyses, gathers 133 experimentally solved fibril structures using ssNMR, solution NMR and cryo-EM from amyloidogenic proteins. STAmP combines all available polymorph structures for a given protein with their APRs. Each entry has a short description indicating mutation type, studied fragment position and method, 3D model and thermodynamic profile. If applicable, it includes annotations about the specific amyloidosis it is found in, the tissue it was derived from and whether it was of human origin. Mutants can be compared using a correlation matrix.

### 2.12 Waltz-DB 2.0

The original Waltz-DB gathered experimentally verified hexapeptides [49]. The newest database iteration, Waltz-DB 2.0 (http://waltzdb.switchlab.org/), expands the original concept with structural information [41]. Currently, it stores 1416 peptide records, 512 of them have amyloid-forming properties and 904 self-assemble into amorphous aggregates. Each entry contains peptide sequence, information on its ability to form amyloid fibrils, source protein identifier and the position of a peptide in its sequence. Moreover, it reports experimental (TEM, ThT assay, FTIR, Proteostat assay) and predicted results (WALTZ, TANGO, PASTA). It also provides computed hydrophobicity and propensity to form amyloid structures, energy calculations and 3D structure predicted by CORDAX [50]. If applicable, Waltz-DB includes a microscopic image of fibril and aggregation kinetics.

### 2.13 Merits and shortcomings of amyloid and aggregation databases

Despite described efforts to provide the community with curated resources on the amyloid formation phenomenon, they all present caveats and limitations given their scope or chosen architecture (Table 2). One of the most prevalent problems is having a limited search engine that hinders finding the desired entries. Another widespread and closely related technical limitation is the limited exporting capabilities of the data into established formats (like CSV or JSON). In addition, some databases limit the amount and type of downloadable information. For instance, AmyLoad allows obtaining entry names, polypeptide sequences, and amyloid propensities in bulk. However, users must access each entry individually if they are interested in the information on experimental procedures or the associated references. Some databases do not provide full dataset download but restrict the data obtention to a single entry, as for CPAD 2.0. The last technical aspect is the difficulty of the database usage, mostly related to the user interface or a way of presenting the data.

**Table 2:** Main limitations of described databases.

Database	Limited filtering	Limited exports	Hard to navigate or use	Limited entry information	Prediction database	Low number of entries
AmyLoad	X	X		X		
AmyloBase	X	X				X
AmyloGraph			X			
Amyloid Atlas	X	X				
AmyPro	X					X
CARs DB	X				X	
CPAD 2.0			X			
Cross-Beta DB						X
Fibrilizer	X	X		X	X	X
PrionScan					X	
StAmP	X					
Waltz DB 2.0						X

The database usability is secondary to the data quality provided by each source. Some databases offer limited information on their entries, either because the individual records are described using very few details or contain missing items. Another important consideration involves databases that provide predicted results. While these resources enable immediate access to the results of predictive algorithms, it is essential to remain mindful of the limitations tied to each predictive algorithm. Finally, several databases gather a low number of entries, often reflecting a focus on a particular topic, like naturally occurring cross-β forming amyloid databases (Cross-Beta DB).

The diversity of data stored in these databases allows for exploring mechanisms of protein aggregation and amyloidosis from different angles. However, this diversity hinders the compilation of available resources into a single knowledge base and the subsequent development of the unified benchmark dataset for predictors of amyloidogenicity. Instead, the tools solve a problem best described by a single available dataset.

One major limitation affecting efforts at predicting amyloidogenicity of proteins and peptides is the unanimous focus on sequences as its sole determinant. Although this process is directly tied to the properties of protein sequences, it is heavily influenced by environmental conditions. Therefore, while it is intractable to perform all experiments *in vivo*, more emphasis should be put on reporting the exact conditions where amyloidogenicity is observed. Recently, the MIRRAGGE initiative (Minimum Information Required for Reproducible AGGregation Experiments) established a standardized framework for reporting protein aggregation experiments [51], aiming to increase the consistency and reproducibility of experimental data and subsequently harmonize descriptions of reported experimental conditions. It is paramount to develop tools to assess whether aggregation occurs in physiologically compatible conditions and predict the influence of environmental factors.

## 3 Other amyloid- and aggregation-related databases

The importance of amyloid self-assembly has led to the creation of several databases that compile extensive information on this complex process. While the databases discussed in Section 2 focus on the biophysical aspects of amyloid aggregation, other resources address different aspects of this issue. Databases such as ALZGENE [52], PDGENE [53], or ALSGENE [54] explore the genetic patterns influencing the pathological amyloid accumulation in AD, PD, and ALS, respectively, reporting genetic association of gene variants or non-synonymous single nucleotide polymorphisms (SNPs) to these disorders. By covering meta-analyses from multiple Genome-Wide Association Studies (GWAS) or association studies, novel genes with roles in these amyloidosis (other than the aggregating amyloid protein) can be established. For instance, an increase of the cleavage of amyloid-beta precursor protein (APP) into the Aβ peptide can be observed due to a SNP in Calcium homeostasis 1 (CALHM1) that causes dysregulation of Ca^2+^ homeostasis [55] and by a combination of low expression levels of Oxysterol-binding protein-1 (OSBP1) and high intracellular cholesterol levels [56].

AL-Base [57] takes a more protein-centric approach while maintaining a clear focus on disease. It is a database of antibody light chain sequences associated with plasma cell dyscrasias, especially immunoglobulin light chain amyloidosis. The database contains almost 5000 nucleotide and protein sequences, categorized by germline (κ, λ) and clinical status.

The αSynPEP-DB [58] was based on the discovery of naturally occurring LL-37 human peptide that was observed to inhibit the aggregation of alpha-synuclein protein (αSyn) [59], [60]. The database gathers 123 biogenic peptides found in PD-relevant tissues predicted to have similar inhibitory potential. These peptides with unique structural information are predicted to bind only to the toxic species of αSyn and hold promising therapeutic potential for PD. Each record has peptide name, inhibitory sequence and length, type (neuropeptide, antimicrobial, food-derived, gut-microbiome), helical score, hydrophobic score, dipole moment, and net charge per residue. Expanded information on the peptide such as predicted cytotoxicity, blood-brain barrier permeability or expression levels can be found in each entry.

Amyloid aggregation could also be considered one of the subfields of general protein aggregation. The Aggrescan3D Model Organism Database (A3D-MODB) [61], built upon predictions of Aggrescan3D 2.0 [62], focuses on protein aggregation. A3D-MODB provides proteome-wide predictions for protein solubility and aggregation properties from the native state for 12 model organisms. Each entry includes a detailed description of the protein's structure and aggregation propensity.

The self-assembly of amyloid fibrils has a low thermodynamic cost, making them useful for nanomaterial development. As a result, some databases focus on collecting data related to amyloid aggregation from a nanotechnology perspective. For example, SAPdb [63] contains 1049 entries of experimentally validated nanostructures formed by tripeptides, dipeptides, and single amino acids. It also provides detailed information about their chemical modifications and experimental conditions. While the data primarily comes from amyloid-related resources like AmyLoad and Waltz-DB, this database presents the information uniquely by filtering based on the size of the self-assembled nanostructure.

## 4 Co-evolution of amyloid databases and prediction of amyloid propensity

**Fig. 2:**
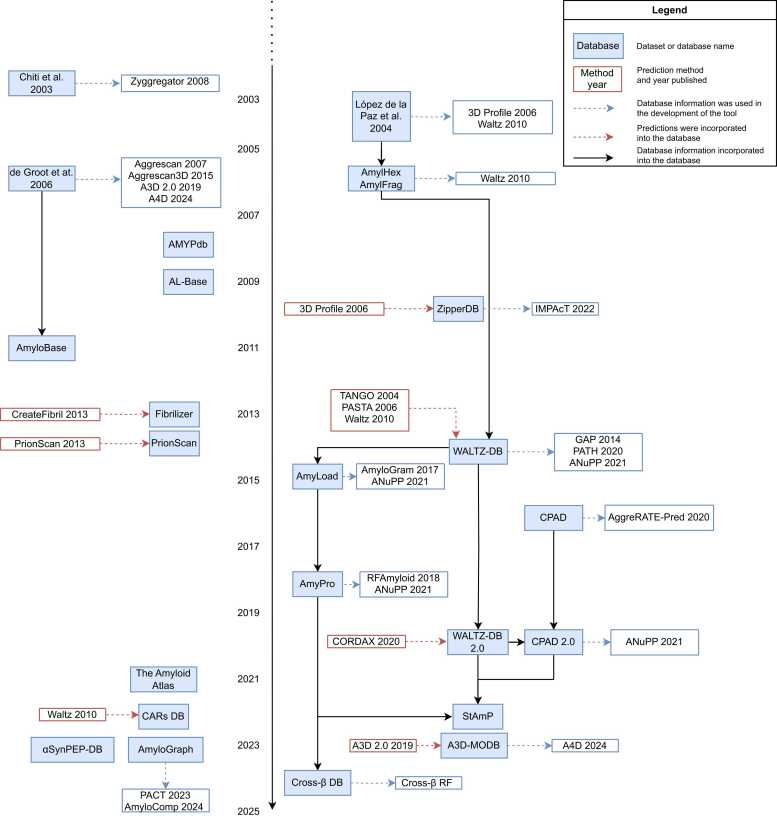
**Timeline of the intertwined relationship between aggregation datasets and databases and predictive tools.** Protein aggregation datasets are differentiated from databases by including the first author and year of publication.

Amyloid self-assembly datasets and databases have been pivotal for pushing forward the understanding of protein aggregation. In part, these resources have paved the way for the development of predictive tools (Fig. 2). Initially, the collection of sequences capable of amyloid self-assembly was motivated by an attempt to disentangle the underlying mechanisms behind this process. In an early study, Chiti *et al.* conducted multiple mutations on the acylphosphatase protein, measuring the changes in aggregation rate *in vitro*, and gathered bibliographical data for seven other polypeptides [64]. The expansion of this initial dataset [65], [66], led to the development of the Zyggregator prediction method [67].

Similarly, López de la Paz and Serrano performed saturation mutagenesis on all positions of an amyloid-forming peptide [68]. The findings of this study and the community-generated AmylHex database [69] spurred the development of the Waltz algorithm, which utilizes position-specific scoring matrices to predict amyloid propensity [70]. The data used to develop Waltz was expanded by an order of magnitude with 1089 experimentally and bibliographically obtained hexapeptides, leading to the development of Waltz-DB. Anew, this expanded dataset facilitated the development of new tools for predicting amyloid aggregation, including CORDAX [50]. The idea of predicting APRs was further refined by the IMPAcT [71], which leverages data from ZipperDB [72] to define the effect of point mutations on the amyloid propensity of proteins.

Correspondingly, the dataset of point mutations of Aβ obtained *in cellulo* [73], later included in AmyloBase, led to the development of AGGRESCAN [46]. Despite this starting point, the newest iteration of this algorithm, Aggrescan4D, predicts the general aggregation propensity of globular proteins considering their 3D structure and the pH of the solution [74], [75].

Even very narrowly specialized datasets enable the development of predictive models. For example, AmyloGraph, a database of amyloid interactions, was used to develop AmyloComp, an algorithm to estimate the structural potential of two sequences to form heterogeneous amyloid fibrils [76] and PACT, dedicated to predicting cross-interactions between amyloid proteins [77].

## 5 Conclusions

The complexity of amyloid aggregation has led to a large information influx, which soon matured into databases. Experimental data and predictions, organized into structured resources, have triggered the development of bioinformatic algorithms for predicting amyloidogenicity and amorphous protein aggregation. These tools have, in turn, accelerated new experimental studies, which have provided data for expanding existing databases and enabling the development of new ones. This cyclic process has significantly improved the understanding of the physicochemical determinants that drive soluble proteins into aggregates, ultimately leading to increased success in developing and formulating protein-based products and therapeutics, anti-aggregation therapies for amyloidosis, and the creation of novel technological applications. Acknowledging the challenges of identifying variables involved in each experiment, we believe that integrating the data stored in these resources will allow the development of highly accurate machine learning-assisted predictive methods, expanding our understanding of the physicochemical determinants that drive proteins into amyloid aggregates.

## CRediT authorship contribution statement

**Valentín Iglesias:** Writing – review & editing, Writing – original draft, Visualization, Data curation, Conceptualization. **Salvador Ventura:** Writing – review & editing, Supervision, Conceptualization. **Michał Burdukiewicz:** Writing – review & editing, Writing – original draft, Visualization, Supervision, Software, Funding acquisition, Conceptualization. **Carlos Pintado-Grima:** Writing – review & editing, Writing – original draft, Conceptualization. **Oriol Bárcenas:** Writing – review & editing, Writing – original draft, Conceptualization. **Jarosław Chilimoniuk:** Writing – review & editing, Writing – original draft, Visualization, Software, Data curation, Conceptualization.

## Declaration of Competing Interest

The authors declare the following financial interests/personal relationships which may be considered as potential competing interests: Valentin Iglesias reports financial support was provided by Polish National Agency for Academic Exchange NAWA. Oriol Barcenas reports financial support was provided by Spanish Ministry of Science and Innovation. Carlos Pintado-Grima reports financial support was provided by Secretariat of Universities and Research of the Catalan Government. Carlos Pintado-Grima reports financial support was provided by European Social Fund. Salvador Ventura reports financial support was provided by Spanish Ministry of Science and Innovation, Catalan Institution for Research and Advanced Studies and Generalitat of Catalunya. Michal Burdukiewicz reports financial support was provided by National Science Centre Poland.
